# P-2199. Impact of Ringer’s Lactate versus Other Fluids in the Hemoconcentration of Children and Adults with Dengue: a Single-Arm Meta-Analysis and Systematic Review

**DOI:** 10.1093/ofid/ofaf695.2362

**Published:** 2026-01-11

**Authors:** Sophia Costa, Leticia R Campos, Gisella Carpi, Jose Luis Boene, Thiago Netto, Oscar Hernández Rios, Taniela M Bes

**Affiliations:** Jose Lucas Municipal Hospital, Belo Horizonte, Minas Gerais, Brazil; Universidade de Ribeirão Preto (UNAERP), Ribeirão Preto, Sao Paulo, Brazil; Hospital de Clínicas de Porto Alegre (HCPA), Porto Alegre, Rio Grande do Sul, Brazil; Eduardo Mondlane University, Maputo, Maputo, Mozambique; Evandro Chagas National Institute of Infectious Diseases (Fiocruz), Rio de Janeiro, Rio de Janeiro, Brazil; Ricardo Palma University, Lima, Lima, Peru; MetroWest Medical Center, Framighan, MA

## Abstract

**Background:**

Dengue fever (DF), an arboviral illness endemic in tropical and subtropical regions, is primarily transmitted by *Aedes aegypti* and *A. albopictus*. Hemoconcentration is a critical marker of disease severity, and fluid resuscitation remains the cornerstone of management. The World Health Organization (WHO) recommends Ringer’s Lactate (RL) as the first-line intravenous (IV) fluid. However, the comparative effectiveness of RL versus other IV fluids in managing hemoconcentration remains under debate.

**Figure 1:**
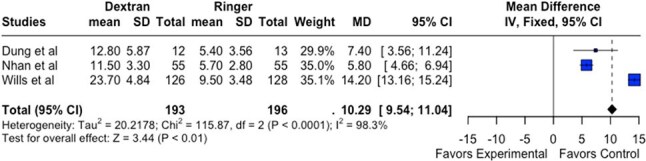
Forest Plot comparing experimental x control Delta hematocrit in 1h - RL x Dextran

**Figure 2:**
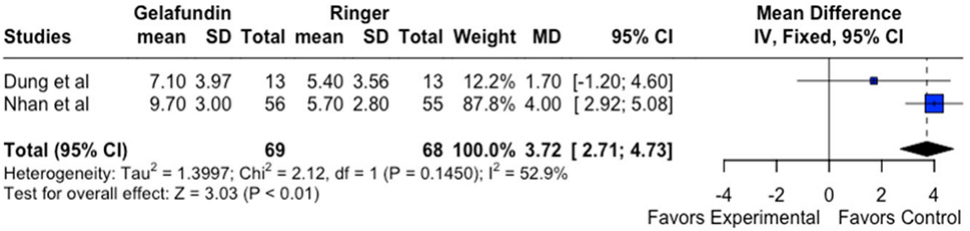
Forest Plot comparing experimental x control Delta hematocrit in 1h - RL x Gelafundin

**Methods:**

We conducted a systematic review and single-arm meta-analysis of randomized controlled trials (RCTs) comparing RL with alternative IV fluids in patients with DF. Databases searched included PubMed, Embase, and the Cochrane Library. The primary outcome was change in hematocrit (ΔHct) at 1 hour post-fluid resuscitation. Secondary outcomes included hospitalization duration and mortality. Meta-analysis was performed using R software. Risk of bias was assessed using the Cochrane ROB-2 tool.

**Figure3:**
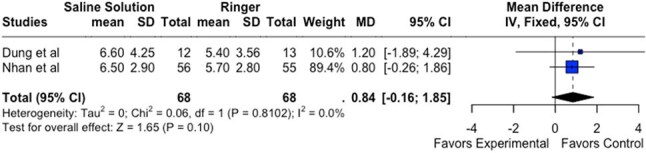
Forest Plot comparing experimental x control Delta hematocrit in 1h - RL x 0.9% Saline Solution

**Results:**

Five RCTs were included:

Dextran vs. RL (n=3 studies): RL was associated with a significantly greater ΔHct (MD = 10.29%; 95% CI: 9.54–11.04; p < 0.01), though heterogeneity was high (I² = 98.3%).

Gelafundin vs. RL (n=2): RL showed a favorable ΔHct (MD = 3.72%; 95% CI: 2.71–4.73; p < 0.01) with moderate heterogeneity (I² = 52.9%, p = 0.15).

0.9% Saline vs. RL (n=2): No significant difference was observed (MD = 0.84%; 95% CI: -0.16 to 1.85; p = 0.10; I² = 0%)

**Conclusion:**

RL demonstrated superior efficacy in improving hemoconcentration compared to Dextran (Figure 1) and Gelafundin (Figure 2). Its performance was comparable to 0.9% saline (Figure 3). These findings support continued use of RL as the WHO-recommended first-line fluid for dengue management. Further trials with standardized protocols are warranted to optimize fluid resuscitation strategies in DF.

**Disclosures:**

All Authors: No reported disclosures

